# Design Thinking in Health Care

**DOI:** 10.5888/pcd15.180128

**Published:** 2018-09-27

**Authors:** Myra Altman, Terry T.K. Huang, Jessica Y. Breland

**Affiliations:** 1 Washington University in St. Louis, St. Louis, Missouri; 2 VA Palo Alto Health Care System, Menlo Park, California; 3 Stanford University, Stanford, California; 4 Center for Systems and Community Design, Graduate School of Public Health and Health Policy, City University of New York, New York, New York

## Abstract

**Introduction:**

Applying Design Thinking to health care could enhance innovation, efficiency, and effectiveness by increasing focus on patient and provider needs. The objective of this review is to determine how Design Thinking has been used in health care and whether it is effective.

**Methods:**

We searched online databases (PubMed, Medline, Web of Science, CINAHL, and PyscINFO) for articles published through March 31, 2017, using the terms “health,” “health care,” or “healthcare”; and “Design Thinking,” “design science,” “design approach,” “user centered design,” or “human centered design.” Studies were included if they were written in English, were published in a peer-reviewed journal, provided outcome data on a health-related intervention, and used Design Thinking in intervention development, implementation, or both. Data were collected on target users, health conditions, intervention, Design Thinking approach, study design or sample, and health outcomes. Studies were categorized as being successful (all outcomes improved), having mixed success (at least one outcome improved), or being not successful (no outcomes improved).

**Results:**

Twenty-four studies using Design Thinking were included across 19 physical health conditions, 2 mental health conditions, and 3 systems processes. Twelve were successful, 11 reported mixed success, and one was not successful. All 4 studies comparing Design Thinking interventions to traditional interventions showed greater satisfaction, usability, and effectiveness.

**Conclusion:**

Design Thinking is being used in varied health care settings and conditions, although application varies. Design Thinking may result in usable, acceptable, and effective interventions, although there are methodological and quality limitations. More research is needed, including studies to isolate critical components of Design Thinking and compare Design Thinking–based interventions with traditionally developed interventions.

## Introduction

Health care systems require continuous innovation to meet the needs of patients and providers (1,2). However, these stakeholders are not always considered when new interventions or system processes are designed, which results in products that remain unused because they do not account for human context, need, or fallibility (3,4). This approach also likely contributes to the decades-long gaps between intervention development and implementation (5). Design Thinking offers a way to close that gap by helping investigators incorporate user needs and feedback throughout the development process.

**Figure 1:**
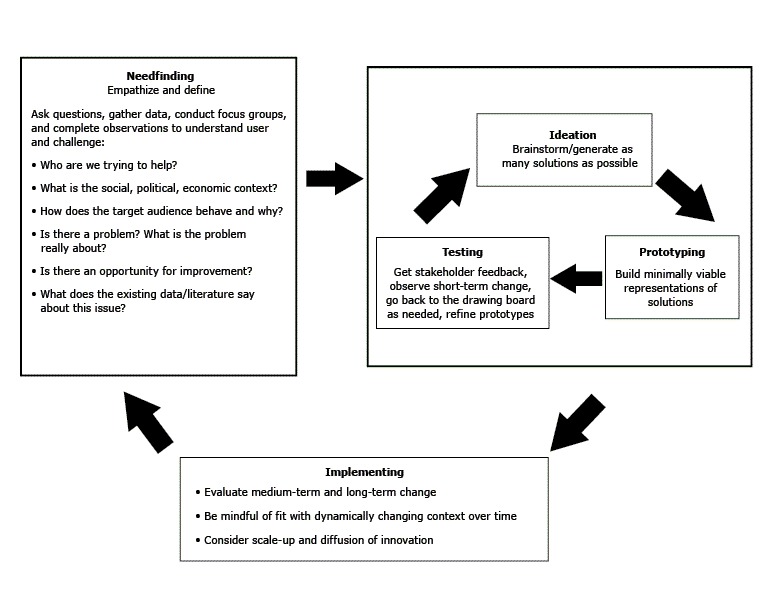
Design Thinking process, stages of design thinking and examples of exercises used and questions asked in each stage, systematic review on Design Thinking in health care, search results through March 31, 2017.

Design Thinking is an approach that prioritizes developing empathy for users, working in collaborative multidisciplinary teams, and using “action-oriented rapid prototyping” of solutions (2,6). It is an iterative process, with innovation emerging only after cycling through several rounds of ideation, prototyping, and testing, which distinguishes it from the traditional linear and often top-down approach to health intervention design (Figure 1) (1,2,4). Design Thinking has been used across sectors to solve complex problems, including the redesign of an elementary school curriculum to enhance student engagement (7), and in domains such as aviation (8) that, like health care, have high levels of risk. Design Thinking is similar to both “user-centered design” and “human-centered design,” which are both referred to as “Design Thinking” in this article.

There is much enthusiasm for the use of Design Thinking in health care, from intervention development to large-scale organizational and systems changes (9). However, health care settings present different challenges than do other domains, so it is important to consider these challenges in assessing whether Design Thinking provides added benefit over traditional approaches. With this in mind, the purpose of this review was to answer the questions, “How has Design Thinking been used to design interventions in health care settings, and have these interventions been effective?”

## Methods

### Data sources

Studies published through March 31, 2017, were identified through searches of online databases (PubMed, Medline, Web of Science, CINAHL, and PyscINFO) using the following search terms: “health,” “health care,” or “healthcare”; and “Design Thinking,” “design science,” “design approach,” “user centered design,” or “human centered design.” Additional articles were included if they were referenced as original research articles in existing articles. To provide an overview of the range of uses of the Design Thinking approach, we did not limit our review to specific populations or conditions and included articles addressing multiple health promotion and disease prevention topics. Given the search terms, the likely target populations for inclusion were patients and health care professionals and the settings in which they work or seek care.

### Study selection

We reviewed selected articles using PRISMA guidelines (10,11) and entered citations into a reference manager, which removed duplicates. To be eligible for inclusion, studies had to be written in English, be published in a peer-reviewed journal, provide outcome data on a health-related intervention, and use Design Thinking in intervention development, implementation or both.

There are multiple definitions of Design Thinking, so we focused on the key principles common to most descriptions of the approach; thus, the list of Design Thinking approaches is not exhaustive. Studies were considered to use Design Thinking if they 1) described user/needs assessment, 2) involved iterative prototyping/testing of the intervention with user feedback, and 3) tested the intervention with target users (2,4). The user/needs assessment could include contextual observation of users in the setting in which they would interact with the innovation, interviews, narrative accounts, and documentation from users, gathering extreme user/outlier stories or a review of existing literature and work (2,6). Prototyping included activities such as creating a series of low-fidelity and high-fidelity prototypes of the potential innovation and refining it multiple times through iterative cycles of feedback from end users, stakeholders, and experts. Testing the intervention with target users included implementing and testing the innovation while continuing to refine it on the basis of user feedback and data (1,2,4). Design Thinking is also similar to other techniques, such as plan-do-study-act cycles and formative evaluations. We considered the emphasis on empathizing with the user and the use of low-fidelity prototyping to be key distinguishing features of Design Thinking, so only articles that explicitly indicate their use of these approaches were included. Initial screening was completed for all selected abstracts, and a second round of screening was completed on eligible full-text articles.

### Data abstraction

Data were collected on target users, health conditions, objective of the intervention, details on the Design Thinking process, study design and sample, and reported health outcomes. If information was not reported in the article, we contacted the study authors. Studies were also evaluated to determine whether the intervention improved all targeted outcomes (successful), at least one targeted outcome (mixed success), or no targeted outcomes (not successful). Data quality was assessed using the National Institutes of Health’s (NIH’s) National Heart, Lung, and Blood Institute Study Quality Assessment Tools (12).

### Study extraction

**Figure 2:**
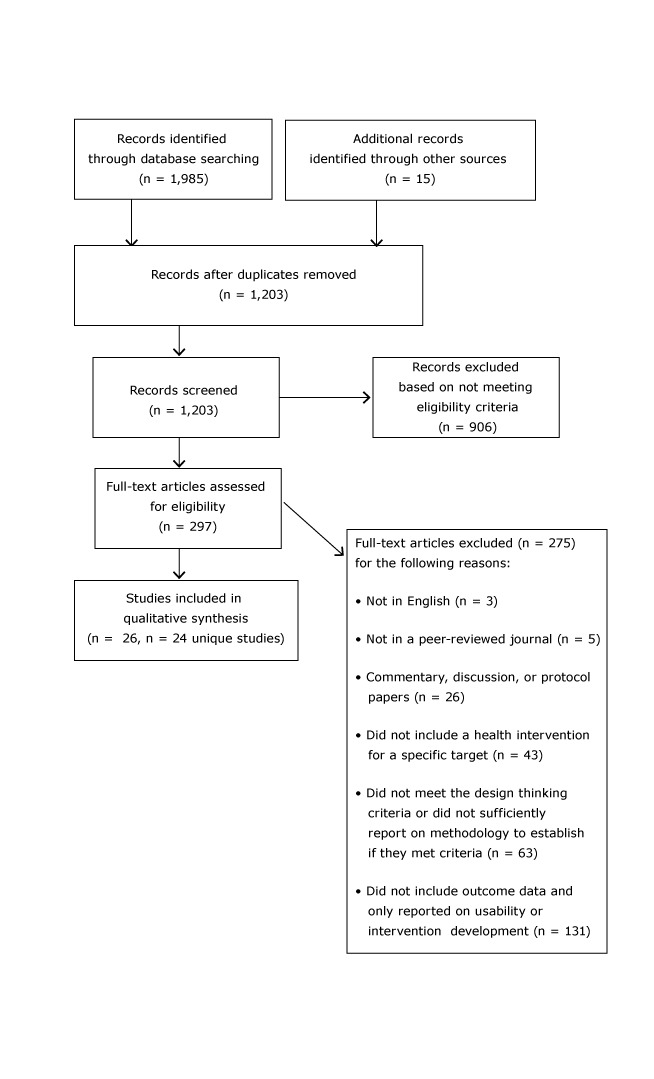
PRISMA 2009 flow diagram, systematic review on Design Thinking in health care, search results through March 31, 2017.

Figure 2 presents study flow based on the PRISMA study guidelines (10,11). After the initial search, the authors separately screened all abstracts based on the eligibility criteria. One author reviewed all full-text articles (N = 297), and a second author reviewed roughly 15% as a reliability check. Agreement on inclusion/exclusion was more than 80%. Any abstracts or articles for which there was disagreement or uncertainty were reviewed by 2 authors and discussed until consensus was reached. A total of 26 papers representing 24 interventions were included in the analysis. Two authors reviewed all included studies.

## Results

### Study characteristics

A summary of all included studies is provided in (Table 1). Eleven studies were successful (13–25), 12 reported mixed success (26–37), and one reported no success (38) (Table 1 and Table 2). Sample sizes of included studies ranged from 12 to 291, but most studies were small; 14 studies had fewer than 40 participants. Eleven (45.8%) used a control group (15,17,18,24,26–29,31,33,35,38,42), and 4 (16.6%) compared a design-thinking intervention to an intervention designed using traditional methods (17,18,24,26,35). Two of the studies included were “good” quality, 13 were “fair” quality, and 9 were “poor” quality. All studies used Design Thinking methodology in intervention development, and 3 also used it for implementation (16,20,25,43)

**Table 1:** Study Characteristics and Design Thinking Methodology, Systematic Review on Design Thinking in Health Care, Search Results Through March 31, 2017

Author, Year	Location	Study Design	Study Quality	Control Condition/Group	Sample Size (No. of Users)	User	Needs Assessment	Low-Fidelity Prototype	Iteration^a^
Adirim et al, 2012 (13)	Canada	Pre/post	Fair	NA	45	Patients	Lit review, expert consultation	DNR	≥3
Anders et al, 2012 (26); Wachter et al, 2003 (37)	Northwestern United States, Southern-central United States	Pre/post	Fair	Conventional tabular display (within-subjects comparison)	32	Providers	Lit review, expert consultation	Yes	3
Cristancho-Lacroix et al, 2014 and 2015 (27,39)	Paris, France	Pilot RCT	Fair	Usual care	49	Caregivers/family members	Lit review, expert consultation	DNR	4
Devito Dabbs et al, 2009a and 2009b (15,40)	Pittsburgh, PA	Pilot RCT	Fair	Standard care	30	Patients	Lit review, surveys, field interviews, and observation of patients	Yes	≥5
Farmer et al, 2017 (28); Velardo et al, 2017 (41)	Oxfordshire and Berkshire, United Kingdom	RCT	Good	Standardized usual care	166	Patients	Lit review	No	DNR
Gilliam et al, 2014 (29)	Chicago, IL	Pilot RCT	Good	Usual care	52	Patients	Lit review, meetings with clinicians and patients	Yes	DNR
Hartzler et al, 2016 (30)	Seattle, WA	Pre/post	Poor	NA	12	Patients/providers	Lit review, focus groups with patients, interviews with patients and providers	Yes	≥2
Kamal et al, 2011 and 2014 (31,42)	Chicago, IL; Vancouver, Canada	Pre/post field study	Poor	No use of intervention in one clinic setting (no control group in other clinic or nonclinical setting)	35	Patients	Theoretical models, patient questionnaires	Yes	3
Koehly et al, 2015 (32)	Washington, DC	Pre/post	Fair	None	40, 45	Caregivers/family members	Lit review, expert consultation	DNR	3
Kuipers et al, 2016 (14)	The Netherlands	Field experiment	Fair	None	37	Providers	Focus groups with nurses, occupational therapists, and caregivers.	Yes	DNR
Lin et al, 2015 and 2011 (16,43)	Northern California	Pre/post	Fair	None	125 nursing units (14 hospitals)	Providers	Field work - observing, shadowing, and interviewing frontline staff	Yes	DNR
Luna et al, 2016 and 2017 (17,18)	Buenos Aires, Argentina	Experimental crossover design	Fair	Alert system with traditional software development	30	Providers	Interviews, contextual observations	Yes	≥3
McGaffey et al, 2010 (19)	Pittsburgh, PA	Pre/post	Fair	None	165	Patients	Lit review, expert consultation, shadowing and interviews with parents and children, focus groups	DNR	≥2
Pottenger et al, 2016 (20)	Washington, DC	Pre/post	Poor	None	22 teams	Providers	Observation, patient and staff interviews	DNR	DNR
Raghu et al, 2015 (21)	Andhra Pradesh, India	Cross-sectional	Fair	None	292 Patients, 14 providers	Providers	Lit review, expert consultation	DNR	≥2
Rizzo et al, 2010 (22); McLay et al, 2012 (44)	San Diego, CA	Pre/post	Fair	None	20	Patients	Expert consultation, patient surveys	DNR	DNR
Sanchez-Morillo et al, 2015 (23)	Cadiz, Spain	Pre/post	Poor	None	15	Patients	Field study, expert feedback	Yes	3
Schoemans et al, 2016 (24)	Leuven, Belgium	Quasi-experimental crossover design	Poor	Standard paper forms	28	Providers	Expert consultation	Yes	2
Trail-Mahan et al, 2016 (25)	Northern California	Pre/post	Poor	None	21 Medical centers	Providers	Interviews, observations, focus groups	DNR	Multiple
van Besouw et al, 2016 (33)	Southampton, UK	Unblinded, randomized crossover design	Poor	Wait list control	21	Patients	Consultation with end users, discussion of existing resources	Yes	DNR
Verwey et al, 2014 (34); van der Weegen et al, 2013 (45)	The Netherlands	Pre/post	Poor	None	20	Patients/providers	Lit review, interviews and focus groups with nurses and patients, expert consultation	Yes	DNR
Welch et al, 2010 and 2013 (38,46)	Indiana	Pilot RCT	Fair	Time-matched PDA app	44	Patients	User needs assessment (did not report details), lit review	Yes	≥2
Wentzel et al, 2016 (35)	The Netherlands	Pre/post	Poor	Regular information sources	34 (n =7 for control comparison)	Providers	Focus groups, onsite observation	Yes	DNR
Yu et al, 2014a and 2014b (36,47)	Toronto	Cohort Study	Fair	None	81	Patients	Lit review	DNR	≥5

Abbreviation: DNR, did not report; NA, not applicable; RCT, randomized control trial.

^a^ If 2 studies are cited, the earlier article is the intervention development methodology and the later article is the evaluation study.

**Table 2:** Study Objectives and Results, Systematic Review on Design Thinking in Health Care, Search Results Through March 31, 2017

Author, Year	Target Condition/ System Process	Intervention Modality	Intervention Objective	Results^a^	Outcomes
Adirim et al, 2012 (13)	Breast cancer	Educational pamphlet	Achieve and maintain bone health in breast cancer survivors	Perceived knowledge increased for both low-income (*P* = .007) and high-income (*P* = .004) respondents	Successful
Anders et al, 2012 (26) Wachter et al, 2003 (37)	ICU patient deterioration	Integrated graphical information display (IGID)	Improve nurses' ability to detect abnormal ICU patient states	Accurate detection of change in patients’ states was higher for the IGID than control (*F*_1,119_ = 13.0, *P* = .02); no difference in perceived workload	Mixed success
Cristancho-Lacroix et al, 2014 and 2015 (27,39)	Caregiver stress	Web-based psychoeducational program	Reduce caregiver stress	No difference in caregiver stress, increased knowledge at 3 months (Cohen’s *d* = 0.79, *P* = .008) but not 6-month follow-up in intervention compared with control group, no differences in self-efficacy, burden, perceived health status, depression	Mixed success
Devito Dabbs et al, 2009a and 2009b (15,40)	Lung transplant	Handheld computer-based intervention	Promote self-care agency, self-care behaviors, and HRQOL in the early months after lung transplantation	Higher self-care agency (*F*_1,27_ = 10.95; *P* = .003; *r* = 0.54), high self-monitoring and spirometry rates, higher likelihood of high adherence and high number of contacts with transplant coordinator (*r* [effect size] for these outcomes, range = 0.45–0.57). Higher HRQOL in intervention compared with control (*r* [effect sizes] for these 3 outcomes, range = 0.41–0.46)	Successful
Farmer et al, 2017 (28); Velardo et al, 2017 (41)	COPD	Internet-linked, tablet computer-based monitoring and self-management support system	Improve quality of life and clinical outcomes	No difference in quality of life in intervention compared with control, intervention groups showed better overall health status (*P* = .30) and fewer visits to general practitioner practice nurses (*P* = .30) compared with control group, no difference on COPD health status, hospital deaths, exacerbations, time to first exacerbation, beliefs about respiratory medicine, medication use, smoking cessation, mood compared with control group	Mixed success
Gilliam et al, 2014 (29)	Contraceptives	App (mobile/tablet)	Improve interest in long acting reversible contraceptive methods	Increased contraceptive knowledge (*P* < .001) and interest discussing the implant (*P* = .02) (not the IUD) in intervention group compared with control group, no difference in selection of contraceptive methods between groups	Mixed success
Hartzler et al, 2016 (30)	Prostate cancer	Clinical dashboard	Display personalized trends in patients’ HRQOL following prostate cancer treatment to facilitate meaningful patient-provider discussion	Greater patient reports of quality indicators with dashboard use (Wilcoxon rank sum tests = 9; *P* < .02). No change in patient self-efficacy, visit satisfaction, or patient–provider communication	Mixed success
Kamal et al, 2011 and 2014 (31,42)	Health behavior	Online Social network	Utilize online social networks to promote determinants of health behavior change	Improvements in some individual-based determinants of health behavior (attitude toward physical activity [*P* = .04], self-efficacy in eating healthy foods [*P* = .04], self-efficacy in performing physical activity [*P* = .04]); no changes in socially based determinants.	Mixed success
Koehly et al, 2015 (32)	Family health history education	Workbook	Educate individuals about their disease risk based on their family history, provide behavioral and screening guidelines based on this risk	High levels of understanding and ability to assess personal disease risk, increased intention and confidence to increase fruit and vegetable and fiber consumption (*P* < .05), no change in intention and confidence to increase physical activity	Mixed success
Kuipers et al, 2016 (14)	Lower back pain in nurses	Serious game	Train nurses in lifting and transfer techniques to prevent lower back problems	Increased play predicted increased game scores (ie, participants moved toward desired behavior (proper lifting technique) with increased play (*b* = .108; *t*_618_ = 23.87; *P* < .001)	Successful
Lin et al, 2011 and 2015 (16,43)	Nursing handoff communication	Systems process change	Improve nursing handoff communication across a hospital system	High rates of intervention spread across system (100% of the 64 medical/surgical units and 47 [77.0%] of the 61 specialty units), improvements in nurse communication (HCAHPS communication scores increased from 73.8% in 2010 to 77.4% in 2014), ratings (HCAHPS score for 82 nursing units across medical centers with comparable data improved from 73.1% [SD, 3.5] in 2010 to 76.4% [SD, 4.9] in the first quarter of 2014 [*P* < .001], and behavior [NKE nursing behavior bundle] improved from 65.9% in 2010 to 71.3% in the first quarter of 2014)	Successful
Luna et al, 2016 and 2017 (17,18)	Drug interaction alerts	Computer interface	Increase the efficiency, effectiveness of an electronic health record drug interaction alert system	The design thinking intervention was more efficient for time (*P* < .001) (not number of clicks or words) and more effective (more error free reports) than the traditional alert system	Mixed success
McGaffey et al, 2010 (19)	Obesity and health behavior	Classroom game	Improve children’s knowledge and beliefs related to obesity and nutrition	Increased knowledge of obesity, nutrition, exercise, and portions (of the 14 questions, 11 questions showed significant (*P* < .01) increases in the percentage of correct responses at either one or both follow-up points compared with baseline; for the 3 questions that did not show a significant increase, most students had correctly answered them preintervention)	Successful
Pottenger et al, 2016 (20)	Care transitions and discharge processes	System process change	Improve patient perception of care transitions and discharge processes	Improvements in patient ratings of discharge information (% of patients giving top scores on HCAHPS Discharge Information increased by at least 3.4%) and care transition ratings (% of patients giving top scores on HCAHPS Discharge Information increased by at least 3.0%)	Successful
Raghu et al, 2015 (21)	Cardiovascular disease	Mobile health tool	A clinical decision support tool to assess and manage cardiovascular disease risk in a resource-constrained setting using minimally trained health workers	Successfully measured risk profile and referred patients to higher level of care	Successful
Rizzo et al, 2010 (22) McLay et al, 2012 (44)	PTSD	Virtual reality	Treatment of PTSD using virtual reality-delivered exposure therapy	Decrease in symptoms of PTSD (*t*_19_ = 5.92, *P* < .001; *t*_16_ = 6.97, *P* < .001), depression (*t*_19_ = 3.69, *P* = .002; *t*_16_ = 4.05, *P* < .001), and anxiety (*t*_19_ = 3.67, *P* = .003; *t*_16_ = 5.36, *P* < .001) in completers between baseline and posttreatment and 3-month follow-up, respectively.	Successful
Sanchez-Morillo et al, 2015 (23)	COPD	Multimodal mobile app	COPD self-monitoring support	High levels of symptom reporting (compliance of 86.1%) and an increase in COPD knowledge	Successful
Schoemans et al, 2016 (24)	GvHD	App (computer/mobile/tablet)	Help clinicians diagnose and score the severity of GvHD faster and more accurately	Significant increase in diagnostic (93% vs 68% correct) and scoring (88% vs 45% correct) accuracy with the design thinking intervention compared with standard forms	Successful
Trail-Mahan et al, 2016 (25)	Pain management	System process change	Improve inpatient pain management	Improvements in patient satisfaction with pain management (HCAHPS pain management composite score from 63.9% to 72.7%, *P* < .05)	Successful
van Besouw et al, 2016 (33)	Hearing loss	App (computer)	Improve music perception in cochlear implant users	Improved instrument recognition (t_6_ = 2.10, *P* = .04, *r* = .65), inconsistent improvements in speech perception, inconclusive results for melodic contour identification, no significant changes in music listening habits, no change in sound quality.	Mixed success
Verwey et al, 2014 (34); van der Weegen et al, 2013 (45)	COPD and diabetes	Accelerometer, app, Internet app	Support patients and nurses in primary care to increase physical activity	Mean physical activity significantly increased (by 10.6 min/d, from 28.7 (SD = 21.1) min/d in the first 2 weeks compared with 39.3 (SD = 24.2) min/d in the last 2 weeks (*P* = .02), did not report changes in sedentary activity	Mixed success
Welch et al, 2010 and 2013 (38,46)	End-stage renal disease	App (mobile)	Electronic application to assist hemodialysis patients in self-monitoring of diet and fluid intake	No differences on interdalytic weight gain, self-efficacy, perceived benefit, or perceived control between groups;	Not successful
Wentzel et al, 2016 (35)	Antibiotic use	Information app	Support nurses in antibiotic stewardship programs	compared with traditional information sources, use of the design thinking intervention showed improvements in perceived information about antibiotics, time to get information (*P* < .001), increased understanding between nurses and providers (*P* = .34), with no changes in openness or accuracy between providers, behaviors, or teamwork	Mixed success
Yu et al, 2014a and 2014b (36,47)	Diabetes	Website — self-management tool	Support self-management of type 2 diabetes mellitus to improve psychological and clinical outcomes	Short-term increase (0.13; 95% CI, 0.06–0.20; *P* < .001), but not long-term increase in self-efficacy; self-care improved long-term (increase of 0.44 [95% CI, 0.23–0.63]; *P* < .001); short-term (−2.29; 95% CI, −3.76 to −0.81; *P* = .002) but not long-term decrease in diabetes distress; no effect on HbA1c, blood pressure, cholesterol, or weight	Mixed success

Abbreviations: App, application; CI, confidence interval; COPD, chronic obstructive pulmonary disease; GvHD, graft vs host disease; HBA1c, hemoglobin A1c; HCAHPS, Hospital Consumer Assessment of Healthcare Providers and Systems; HRQOL, health-related quality of life; IGID, integrated graphical information display; IUD, intrauterine device; NKE, nurse knowledge exchange plus; PTSD, posttraumatic stress disorder; SD, standard deviation.

^a^ All results significant at *P* < .05.

The 24 included interventions targeted a range of conditions, including 19 related to physical health (17 unique conditions), 2 related to mental health, and 3 related to systems processes. Approximately two-thirds of the interventions were mobile telephone–based or tablet-based.

### Summary of findings by target user

**Patient-facing interventions (n = 11).** Five interventions were successful: 4 with a pre/post design (13,19,22,23,44) and 1 pilot randomized control trial (RCT) (15). Five reported mixed success, including one pre/post design (31), one pilot RCT (29), one RCT (28), one cohort study (47), and one unblinded, randomized crossover design (33). One, a pilot RCT, was not successful (46).

**Provider-facing interventions (n = 9).** Six were successful, including 3 studies using a pre/post design (16,20,25), one field experiment (14), one using a quasi-experimental crossover design (24), and one cross-sectional study (21). Three had mixed success, including 2 studies with an experimental crossover design (17,18,26) and one with primarily a pre/post design, one portion of which was a randomized crossover design (35).

**Patient-facing and provider-facing interventions (n = 2).** Both reported mixed success and were pre/post designs (30,34,45).

**Caregiver-facing or family-facing interventions (n = 2).** Both reported mixed success, one in a pilot RCT (27,39) and one using a pre/post design (32).

**Summary of Randomized-Controlled Trials (K = 5).** Of the RCTs and pilot RCTs reviewed, one demonstrated success on all outcomes (15,40), 3 showed mixed success (27–29,39,41), and one reported no enduring significant results (38,46).

### Summary of studies directly testing Design Thinking methodology

Four studies directly compared interventions created with Design Thinking to interventions created with traditional methods. In one study with a within-sample experimental crossover design (26), a Design Thinking–based graphical information display to improve nurses’ ability to detect changes in patients’ physiological states in an intensive care unit (ICU) was compared with a conventional display in commercial, electronic ICU charting systems. The Design Thinking intervention resulted in improved detection of changes in patient states and greater ease of use, usefulness, satisfaction, and support of understanding, but no differences in workload for nurses (26). Another study using an experimental crossover design compared 2 computer interfaces designed to display drug interaction alerts, one developed using Design Thinking and one using traditional software (17,18). Whereas the design of the traditional software was not described, the traditional display included only basic text information. In this study, users (ICU nurses) were more efficient and effective, and reported higher satisfaction with the Design Thinking interface. Another study using a quasi-experimental crossover design used Design Thinking to develop an application to guide clinicians in detecting and scoring the severity of graft versus host disease (GvHD) (24). When compared with paper-based NIH guidelines, users of the application (app) signficantly improved diagnostic and scoring accuracy. A final study compared a Design Thinking–based app that provided nurses with information about antibiotic use with regular information sources (which were not described) (35). In the randomized portion of this study, nurses using the app found information on antibiotic use more quickly; however, the app did not enhance their ability to improve antibiotic-related behaviors. (Only 7 participants were included in the randomized portion of the study.) Whereas the development of the control intervention was not fully described in these papers, based on the limited descriptions given, it is likely that it did not include key elements of Design Thinking such as user feedback and prototyping.

## Discussion

The 24 interventions summarized in this review provide an overview of the breadth of Design Thinking's applicability in health care and demonstrate that it is feasible and applicable to multiple health care domains. It has been applied across a range of diverse patient populations and conditions, including chronic obstructive pulmonary disease (28,34), diabetes (34,47), caregiver stress (27), and posttraumatic stress disorder (22). It also has been applied to systems process changes, such as nursing handoffs (16) and drug–drug interaction alerts (17,18). Results also demonstrate that, although it is often applied to electronic interventions, Design Thinking is feasible for use in other modalities (eg, on paper, in person).

Initial results of the interventions included in this review are promising; all but one demonstrated positive effects on at least one identified outcome, and half showed positive effects on all measured outcomes. In addition, in the studies that directly compared the Design Thinking intervention with a traditional intervention, the Design Thinking intervention generally demonstrated improved outcomes and higher usability and satisfaction.

However, none of these studies were RCTs with large sample sizes. Design Thinking interventions have been tested primarily in pre/post designs or pilot RCTs with small samples. Furthermore, most studies included were poor or fair quality, with only 2 being considered good quality. Importantly, the criteria used to assess quality were based on traditional research approaches, and many of the features of poor-quality studies were included by design; some had small sample sizes to generate insights and to test assumptions rapidly, and some were pilot studies. This feature of Design Thinking also may account for the limited use of large RCTs; however, this poses a challenge when evaluating the effectiveness of the approach. More work in this area using more rigorous methods and larger samples is critical to fully understanding the benefits of Design Thinking. Although many studies that used Design Thinking were excluded from our review because they did not include sufficient outcome data (n = 131), full-scale trials of many of these interventions are under way, results of which will provide more evidence about the effectiveness of this approach in health care. In addition, no studies measured Design Thinking directly to explain how or what components of Design Thinking lead to improved usability and effectiveness, limiting the field’s ability to disseminate the most effective components and refine the Design Thinking approach for health care.

Design Thinking methods varied among the studies reviewed. For example, only 6 studies conducted contextual observations of users during the needs assessment phase, no studies reported a brainstorming stage, 10 studies did not use low-fidelity prototypes, and some reported a small number of iterations (eg, one mixed-success trial had 4 intervention iterations, but only 2 iterations were evaluated with target users [27]). Using more thorough and structured Design Thinking methodology may have resulted in more consistent and enhanced outcomes. At the same time, Design Thinking is meant to be flexibly applied. Future work should balance that flexibility with the potential benefits of a more systematic approach.

Our results suggest that one area where Design Thinking could be especially useful is in designing interventions for underserved populations whose needs may be overlooked by other approaches. For example, the study of a mobile health tool for detecting and managing cardiovascular disease in rural India required significant feedback from the end users — minimally trained health workers — to ensure that the intervention was suited to their level of technological familiarity as well as the inconsistent technical infrastructure (eg, creating a one-touch navigation system) (21). Using Design Thinking allowed the multidisciplinary team to question assumptions and biases and develop an intervention that was successful, acceptable, and feasible to the actual users, an outcome that may not have been possible using traditional methods (21). Another study evaluated the impact of an education tool to enhance long-acting contraceptive use in a clinic serving mostly African American patients who were included early in the usability testing process to ensure the tool met their needs. Several changes were made as a result, such as including more peer testimonials, which likely increased the tool’s impact and relevance (29). In this way, Design Thinking could also pair well with other approaches that prioritize the inclusion of users in service of reducing health disparities, such as community-based participatory research (48).

### Tensions when using Design Thinking in health care

In their text and through our analysis, the studies included in this review show several challenges to consider when applying Design Thinking to health care. First, there is the possibility of tension between what users want and what providers and researchers believe to be beneficial based on research and expertise (49). Whereas in industry, where an innovation designer may prioritize customers’ preferences to maximize profits, in health care a balance must be struck between creating interventions that are effective and sufficiently palatable and feasible so that they will be used by providers and patients.

Second, tension may exist between the needs assessment, a fundamental step of Design Thinking, and existing literature and evidence base for some conditions. That is, given the evidence, intervention developers may not be willing or see it necessary to conduct their own needs assessment using observation or interview strategies or to brainstorm creative solutions. Indeed, 7 of the studies included in this review reported literature reviews, and possibly expert consultation, as their only needs assessment steps, and none reported brainstorming. One way to overcome this tension is to view evidence as a set of design constraints in which needs assessment, brainstorming, ideation, and prototyping should occur.

A third possible tension relates to balancing the Design Thinking approach of understanding the narrative of outliers with traditional health research methods that prioritize statistics on large samples to produce generalizable results. Conclusions drawn from small user samples should be tested in broader populations to ensure their applicability. Mixed-methods approaches that use both strategies may reduce this tension. For example, a research team that uses a qualitative Design Thinking approach early in the research process (eg, user observations, focus groups, and usability tests with small groups of target users) may be able to generate insights into the key needs of the target population. This approach may also find ways to address these needs, and subsequent quantitative testing of the developed interventions in broader samples will allow the group to evaluate whether their assumptions generalize to the broader population, and the intervention will be more effective as a result.

Fourth, there is inherent tension between a central philosophy of the prototyping process in Design Thinking — to rapidly move through low-fidelity then high-fidelity iterations to fail early and often to more quickly reach a better design (50) — and the risk of serious negative outcomes due to health care failures (eg, death). Many of the studies did not use low-fidelity prototyping or multiple rapid iterations, perhaps because of this tension. However, although there may be some reluctance to experiment with low-fidelity prototypes in health care where morbidity and mortality are at stake, there are low-stakes approaches to low-fidelity prototyping that may minimize risk and improve the pace of innovation (eg, storyboards to illustrate a new clinic process).

### Intervention development and implementation: case example

Considering the role of Design Thinking is important, not only in efficacious intervention development but also in effective implementation into practice (5). Only 3 of the included interventions addressed implementation, but this limited implementation provides insights. For example, in designing a new process for facilitating nurse handoffs between shifts, Lin and colleagues conducted an extensive 6-month intervention development design process that was user-focused and empathic and had rapid iteration in pilot sites (43). However, despite this strong preliminary work, the intervention was not readily accepted when implemented in other clinics. As a participant stated:

After the concepts had been co-developed and field tested with our pilot units . . . we assumed the units were “bought in” to the idea of the change. . . . Surprisingly, our approach to the training resulted in criticism and created skepticism [at other clinics]. . . . They attributed this to “not made here” sentiments from those units not involved in the original design.

To overcome this tension, the team involved additional stakeholders to develop a more user-centered process for the implementation of their Design Thinking innovation, after which they successfully implemented the innovation across 125 nursing units in 14 hospitals over 2 years (16). This study highlights the importance of understanding the context of the setting and users, both when developing and implementing an intervention using a Design Thinking approach. It should also be noted that this process required significant time and energy from stakeholders. One stakeholder commented, “Don’t get me wrong. What we did was fantastic. But it took a lot out of us” (43). This study highlights the importance of staying true to the user-centered nature of Design Thinking throughout the process — from development to implementation — to maximize implementation success. It also highlights the challenges in using this approach. Teams using Design Thinking should be prepared for a more intensive process than traditional, less iterative and user-centered methods.

### Limitations

Given the varied outcomes included in the review and the inconsistent reporting of qualitative outcomes it was difficult to make comparisons across studies. The range of study types and limited number of large scale RCTs testing intervention effects also made it difficult to draw definitive conclusions about effectiveness. At the same time, given that there was only one study with a null result, there was likely publication bias, which may have led to overestimation of the effectiveness of Design Thinking. It is also possible that investigators used methods but did not report them (eg, prototyping). In addition, we did not assess the use of Design Thinking in other health care areas where it may be beneficial, such as the design of physical spaces. Finally, Design Thinking–based health care innovations that were developed and implemented outside research contexts may exist and are thus not reported in the literature.

### Conclusions

Design Thinking is being used in varied health care settings and health conditions, and more studies are forthcoming. This review suggests that Design Thinking may result in more usable, acceptable, and effective interventions compared with traditional expert-driven methods. However, there is inconsistent use of the methodology and significant limitations inherent in the studies, which limits our ability to draw conclusions about this approach. Future studies may benefit from focusing on comparing interventions developed using Design Thinking methods with traditionally developed interventions, including those with RCT designs, and identifying the most useful components of Design Thinking methods. 

Overall, Design Thinking is a promising approach to intervention development, implementation, and dissemination that may increase the acceptability and effectiveness of health care interventions by actively engaging patients and providers in the design process and rapidly iterating innovation prototypes to maximize success.
